# Anti-Inflammatory Effects of Dimethyl Fumarate in Microglia via an Autophagy Dependent Pathway

**DOI:** 10.3389/fphar.2021.612981

**Published:** 2021-05-07

**Authors:** Young-Sun Lee, Deepak Prasad Gupta, Sung Hee Park, Hyun-Jeong Yang, Gyun Jee Song

**Affiliations:** ^1^ Department of Medical Science, College of Medicine, Catholic Kwandong University, Gangneung, Korea; ^2^ Translational Brain Research Center, International St. Mary’s Hospital, Catholic Kwandong University, Incheon, Korea; ^3^ Department of Integrative Biosciences, University of Brain Education, Cheonan, Korea

**Keywords:** neuroinflammation, autophagy, microglia, DMF, anti-inflammation

## Abstract

Dimethyl fumarate (DMF), which has been approved by the Food and Drug Administration for the treatment of relapsing-remitting multiple sclerosis, is considered to exert anti-inflammatory and antioxidant effects. Microglia maintain homeostasis in the central nervous system and play a key role in neuroinflammation, while autophagy controls numerous fundamental biological processes, including pathogen removal, cytokine production, and clearance of toxic aggregates. However, the role of DMF in autophagy induction and the relationship of this effect with its anti-inflammatory functions in microglia are not well known. In the present study, we investigated whether DMF inhibited neuroinflammation and induced autophagy in microglia. First, we confirmed the anti-neuroinflammatory effect of DMF in mice with streptozotocin-induced diabetic neuropathy. Next, we used *in vitro* models including microglial cell lines and primary microglial cells to examine the anti-inflammatory and neuroprotective effects of DMF. We found that DMF significantly inhibited nitric oxide and proinflammatory cytokine production in lipopolysaccharide-stimulated microglia and induced the switch of microglia to the M2 state. In addition, DMF treatment increased the expression levels of autophagy markers including microtubule-associated protein light chain 3 (LC3) and autophagy-related protein 7 (ATG7) and the formation of LC3 puncta in microglia. The anti-inflammatory effect of DMF in microglia was significantly reduced by pretreatment with autophagy inhibitors. These data suggest that DMF leads to the induction of autophagy in microglia and that its anti-inflammatory effects are partially mediated through an autophagy-dependent pathway.

## Introduction

Microglia, innate immune cells that play protective roles against invading pathogens in the central nervous system (CNS) (Afridi et al., 2020; Xu et al., 2020), comprise approximately 10% of all cells in the healthy mammalian brain (Augusto-Oliveira et al., 2019). Microglial activation is observed during viral or bacterial infection, neurodegenerative diseases such as Alzheimer’s disease, and traumatic brain injury (Mariani and Kielian, 2009; Donat et al., 2017; Edison, 2020; Severini et al., 2021); activated microglia can be further subdivided into classical (M1) and alternative (M2) microglia (Jiang et al., 2020). The presence of interferon-gamma and lipopolysaccharide (LPS) switches microglia to the M1 state, wherein they express and/or release interferon-gamma, tumor necrosis factor-alpha (TNF-α), interleukin (IL) 1β, IL-6, IL-12, IL-23, and inducible nitric oxide (NO) synthase (iNOS); microglia in the M1 state have been linked to reduced phagocytic ability. On the other hand, IL-4, IL-10, and IL-13 induce the microglial switch to the M2 state, wherein they release anti-inflammatory cytokines such as IL-4, IL-10, IL-13, and transforming growth factor-beta and increase the expression levels of arginase-1, brain-derived neurotrophic factor, and Ym1; the M2 microglia are recognized for their increased neuroprotective capacity. Thus, M1 and M2 microglia are involved in pathogenic and neuroprotective responses, respectively (Song et al., 2016; Song and Suk, 2017; Gupta et al., 2020).

Autophagy removes unnecessary or dysfunctional proteins and damaged organelles via the lysosomal machinery. Autophagy is critical for the maintenance of neuronal function and is considered to provide neuroprotection in neurodegenerative disorders. Proper regulation of autophagy is associated with beneficial outcomes in neurodegenerative diseases (Nixon, 2013; Cho et al., 2020). Although the interaction between autophagy and inflammation is complicated and controversial, neuroinflammation is implicated in autophagic dysfunction. For example, recent studies have reported neuroprotective as well as anti-neuroinflammatory effects of several autophagic modulators such as rapamycin, metformin, and resveratrol (Sarkar et al., 2009; Thellung et al., 2019).

Dimethyl fumarate (DMF) is a potent anti-inflammatory mediator which has been used for the treatment of psoriasis for many years (Altmeyer et al., 1994). Importantly, DMF has also been approved by the US Food and Drug Administration as an orally bioavailable compound for the treatment of multiple sclerosis (Hoefnagel et al., 2003; Fox et al., 2012; Gold et al., 2012; Xu et al., 2015). DMF reduces proinflammatory responses by inhibiting the NF-κB signaling pathway. In addition, DMF provides neuroprotection against α-synuclein toxicity via the activation of autophagy in a mouse model of Parkinson’s disease (Lastres-Becker et al., 2016). However, there is limited information on the interaction between the anti-inflammatory effect and autophagy activation in DMF-treated microglia. Therefore, we investigated whether DMF induced autophagy in microglia and if DMF-induced autophagy had an impact on the inflammatory activation of microglia.

## Materials and Methods

### Animals

C57BL/6 mice were obtained from Orient Bio (Gyeonggi, Korea) and maintained in specific pathogen-free conditions under a 12 h light:12 h dark light period in the animal facility of Gachon University. All animal experiments were performed under a protocol approved by the Institutional Animal Care and Use Committee at the Gachon University. Eight-week-old male C57BL/6 mice were injected with streptozotocin (STZ) (100 mg/kg intraperitoneal [i.p.], Sigma-Aldrich, Saint-Louis, MO) or PBS, as a vehicle control, after overnight fasting. The tail blood of mice was monitored for the development of hyperglycemia using a glucometer. After confirming that the blood glucose level was over 300 mg/dl for three consecutive days, the mice were injected with DMF (45 mg/kg, i.p.) or PBS, as a control, once a day for four weeks based on the previously published information (Yao et al., 2016; Hayashi et al., 2017).

### Cells

HAPI (highly aggressively proliferating immortalized) cells, a rat microglial cell line, were cultured in Dulbecco's Modified Eagle medium (DMEM) supplemented with 10% fetal bovine serum (FBS) and 100 U/mL penicillin/streptomycin. BV-2 cells, an immortalized murine microglial cell line, were maintained in DMEM supplemented with 5% FBS and 100 U/mL penicillin/streptomycin. Primary mixed glial cells (MGC) were prepared from neonatal C57BL/6 mice on postnatal days 1–3, as previously described (Gupta et al., 2020), with minor modifications. The cell suspensions obtained from brain tissue dissection were cultured with DMEM supplemented with 10% FBS and 100 U/mL penicillin/streptomycin for three weeks, with medium changed every three days. After three weeks, the MGC were harvested, centrifuged, and replated in 96-well (4 × 10^4^/well), 24-well (5 × 10^4^/well), or 12-well (2 × 10^5^/well) plates. For primary microglia isolation from MGC, confluent MGC were treated with mild trypsin (20–25%) for 20 min at 37°C, which resulted in the detachment of an intact layer of cells comprising virtually all astrocytes, leaving undisturbed a population of microglia on the plate. The purity of primary microglial culture was 96.9 ± 0.409%, which was determined by Iba-1immunostaining (Supplementary Figure S1). After washing with phosphate-buffered saline (PBS), the microglia were treated with trypsin (100%) for 5 min at 37°C. The cells were harvested, and the centrifuged cells were replated onto 24 well plates (5 × 10^4^/well) containing poly-D-lysine-coated glass coverslips (diameter, 12 mm) (Saura et al., 2003).

### Measurement of Nitric Oxide Production and Cell Viability

Cell culture medium was harvested after treatment with or without LPS (100 ng/ml) in the presence or absence of DMF. NO production was analyzed by the colorimetric Griess assay to determine the concentration of nitrite, a stable NO metabolite. The absorbance was detected at 540 nm by a microplate reader. Cell viability was determined by the MTT [3-(4, 5-dimethylthiazol-2-yl)-2, 5 diphenyl tetrazolium bromide] assay, and the sample absorbance was detected at 570 nm.

### Real-Time Quantitative Polymerase Chain Reaction and Reverse Transcription-Polymerase Chain Reaction

Total RNA was isolated using Trizol^®^ reagent (Invitrogen, Carlsbad, CA), and cDNA was synthesized using the Moloney murine leukemia virus reverse transcriptase (Promega) and oligo (dT) primers. Real-time quantitative PCR (RT-qPCR) was performed using a 7900HT fast real-time PCR system (Applied Biosystems, Carlsbad, CA) using the following conditions: 95°C for 5 min, followed by 40 cycles at 95°C for 15 s and 60°C for 1 min. As an internal control, cyclophilin mRNA was amplified. The sequences of primer pairs are provided in Supplementary Table S1. Relative copy numbers were determined using the threshold crossing point (Ct) in combination with the ΔΔCt values, calculated by the 7900HT fast real-time PCR software. Traditional reverse transcription-PCR was performed with specific primer sets (Supplementary Table S1) at annealing temperatures of 57–60°C for 24–29 cycles using a T100 Thermal Cycler (Bio-Rad, Richmond, CA). After PCR, 10 µL of each PCR product was electrophoresed on a 2% agarose gel. DNA fragments were detected under ultraviolet light following ethidium bromide staining.

### Western Blot Analysis

Total cell lysates were prepared using ice-cold lysis buffer (150 mM NaCl, 50 mM Tris, 1% Nonidet P-40 and 0.1% sodium dodecyl sulfate). The lysates were separated on sodium dodecyl sulfate-polyacrylamide gels and transferred to polyvinylidene difluoride membranes (Bio-Rad, CA). The membranes were blocked with 5% skim milk for 1 h and incubated with mouse anti-arginase-1 (1:1,000 dilution; BD Biosciences, NJ), rabbit anti-microtubule-associated protein light chain 3 (LC3; 1:1,000 dilution; MBL, Woburn, MA), or mouse anti-β actin (1:5,000 dilution; Sigma-Aldrich) antibody overnight at 4°C. Next, the membranes were incubated with appropriate horseradish peroxidase-conjugated secondary antibodies (1:2000 dilution) for 1 h at room temperature. The blots were visualized with a chemiluminescence detection kit (SuperSignal™ West Femto; Thermo Fisher, Franklin, MA).

### Phagocytosis Assay

Phagocytosis assay was performed with Fluorescent zymosan Bioparticles from *Saccharomyces cerevisiae* (pH-sensitive pHrodo^TM^ Red dye conjugates; Life Technologies, Carlsbad, USA). In brief, primary microglial cells were seeded at a density of 5 × 10^4^ cells/well in 24-well plates and cultured for 48 h in DMEM supplemented with 10% FBS, followed by treatment with 10 μM DMF for 24 h. And then, cells incubated in serum-free medium containing fluorescently labeled zymosan particles (10 μg/ml) for 2 h at 37°C (Grozdanov et al., 2014). The cells were washed three times with PBS and fixed with 4% paraformaldehyde. Fluorescent images were captured using a laser scanning confocal fluorescent microscope (Nikon ECLIPSE 80*i*; Nikon, Japan).

### Immunofluorescent Staining

Immunofluorescent staining of spinal cord sections and fixed cells in culture were performed as previously reported (Song et al., 2019). Briefly, tissue sections or cells fixed with 4% paraformaldehyde were blocked with 1% bovine serum albumin or normal serum in 0.3% Triton X-100 for 60 min at room temperature. The samples were next incubated with rabbit anti-glial fibrillary acidic protein (GFAP; 1:500, DAKO, Glostrup, Denmark), goat anti-Iba-1 (1:200, Novus, CO), or rabbit anti-LC3 (1:500, MBL) antibody in PBS containing 1% BSA at 4°C overnight. The samples were washed three times with 0.3% Triton X-100 in 0.1 M PBS, followed by incubation with FITC- or Cy3-conjugated secondary antibodies (1:200 for tissue sections and 1:500 for fixed cells) for 60 min at room temperature. Fluorescent images were captured using the Nikon ECLIPSE 80*i* laser scanning confocal fluorescent microscope. For the analysis of the immunofluorescence staining, the spinal cord images were outlined the size for the standardized reason of interest (ROI) by ImageJ. The threshold was adjusted and standardized for each image. The mean intensity of the fluorescence in pixels was measured. The percentage of area was calculated by dividing the pixel number of the protein expression with the total unfiltered pixel number in the ROI. For the analysis of LC3 puncta area in HAPI cells, the area of a single cell was marked and the LC3 punctate area was measured using ImageJ. The number of zymosan particles or LC3 puncta was analyzed using particle analyzer in ImageJ (Fiji, version 1.53c, National Institutes of Health, United States) (Supplementary Figure S2).

### Statistical Analysis

Data were presented as means ± standard error of the mean (SEM) from three or more independent experiments. The normality of values had been checked then used parametric or non-parametric test. Statistical significance was analyzed by paired or unpaired Student’s *t*-test for comparison of two groups and one-way or two-way ANOVA followed by multiple comparison test for multiple groups. A *P* value of <0.05 was considered to indicate statistical significance.

## Results

### Dimethyl Fumarate Reduces Gliosis in the Spinal Cord of Streptozotocin (STZ)-Treated Diabetic Neuropathy Mice

**FIGURE 1:**
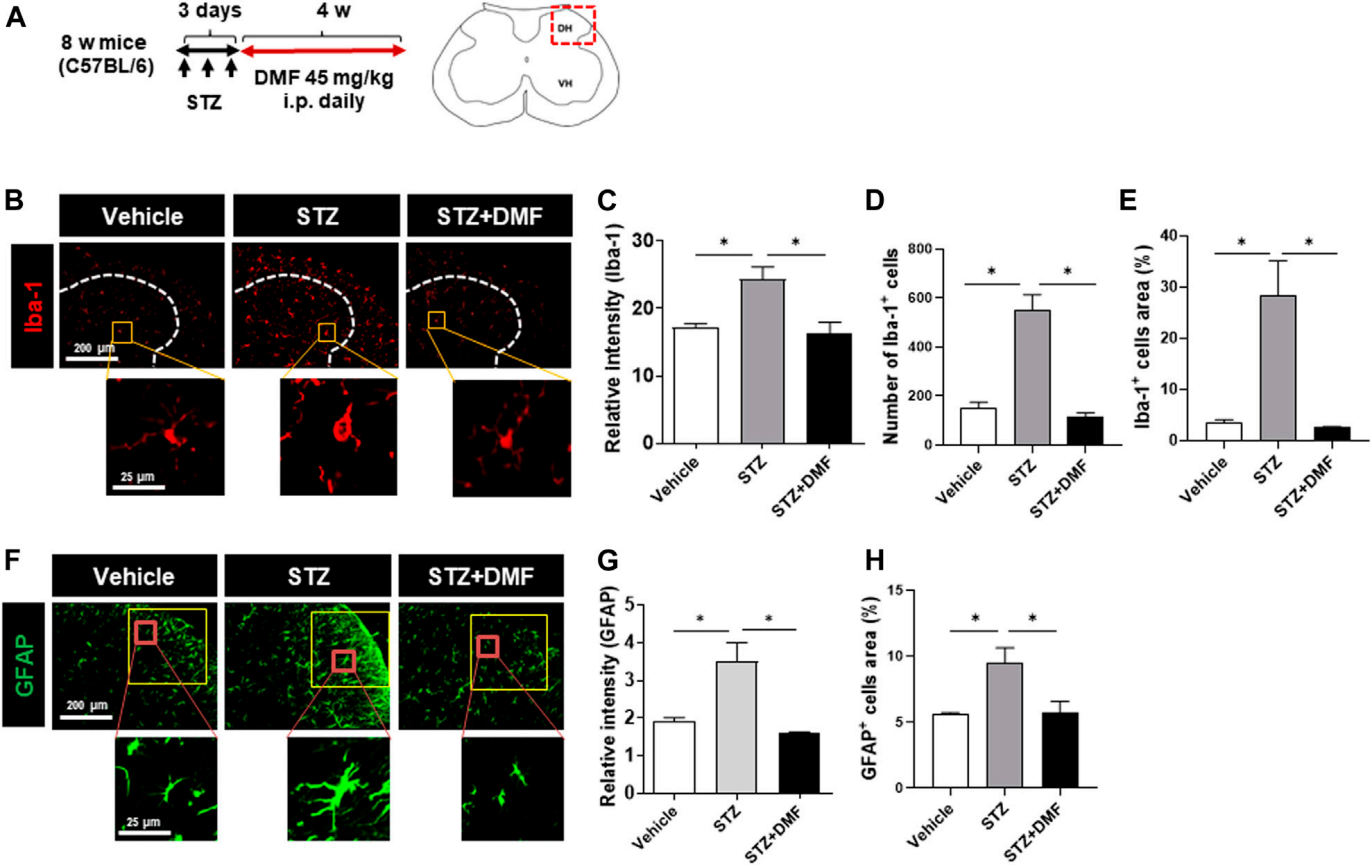
Dimethyl fumarate reduces gliosis in the spinal cord of mice with STZ-treated diabetic neuropathy **(A)** C57BL/6 mice were injected with STZ (100 mg/kg, i.p.). After 3 days, the mice that developed diabetes were administered dimethyl fumarate (DMF, 45 mg/kg i.p.) once a day for four weeks. At the end of treatments, spinal cord sections were prepared and stained with anti-Iba-1 **(B–E)** or anti-glial fibrillary acidic protein (GFAP) antibody **(F–H)**. White dotted lines represent the dorsal horn of the spinal cord. Representative Iba-1^+^ microglia are shown in the magnified images. The quantified relative fluorescence intensity for Iba-1^+^ cells **(C)**, number of Iba-1^+^ cells **(D)**, and the area of Iba-1^+^ cells in the spinal cord **(E)** were analyzed. Representative GFAP^+^ astrocytes are shown in the magnified images **(F)**. The quantified relative fluorescence intensity for GFAP^+^ cells **(G)** and the area of GFAP^+^ cells in the spinal cord **(H)** were analyzed (*n* = 3 mice/group, 3 images/mouse). Data are presented as means ± standard error of the mean (SEM). *P < 0.05 compared with STZ-treated mice from ANOVA, Tukey’s multiple comparison test.

STZ-induced diabetic neuropathy is a well-defined mouse model of neuroinflammation and reactive microglial activation (Wodarski et al., 2009; Talbot et al., 2010; Ellis and Bennett, 2013). To investigate whether DMF administration inhibited the hyperactivation of microglia and astrocytes, we measured the levels of Iba-1 and GFAP in the spinal cord of mice with STZ-induced diabetic neuropathy. The relative Iba-1 intensity was significantly higher in the STZ-treated mice than in the untreated control mice (*P* < 0.05) as shown in Figures 1B,C. The number and the area of Iba-1 positive cells also significantly increased in the STZ-treated mice than in the control mice, however the DMF treatment significantly lowered the intensity, number and area of Iba-1 positive cells in the spinal cord compared to STZ-treated mice (*P* < 0.05) (Figures 1C–E). Similarly, we found astrogliosis in STZ-treated mice (Figures 1F–H). STZ-induced astrogliosis was significantly reduced by DMF treatment as analyzed by the reduced intensity and the area of GFAP-positive cells. Notably we found hyper-ramified and amoeboid shaped microglia in STZ-treated mice. In contrast, the microglia morphology in DMF-treated mice showed small cell bodies and thin processes as in control group. These results suggested that DMF suppressed the activation of microglia and astrocytes in the spinal cord of mice with STZ-induced diabetic neuropathy.

### Dimethyl Fumarate Downregulates the Inflammatory Activation of Microglia

**FIGURE 2:**
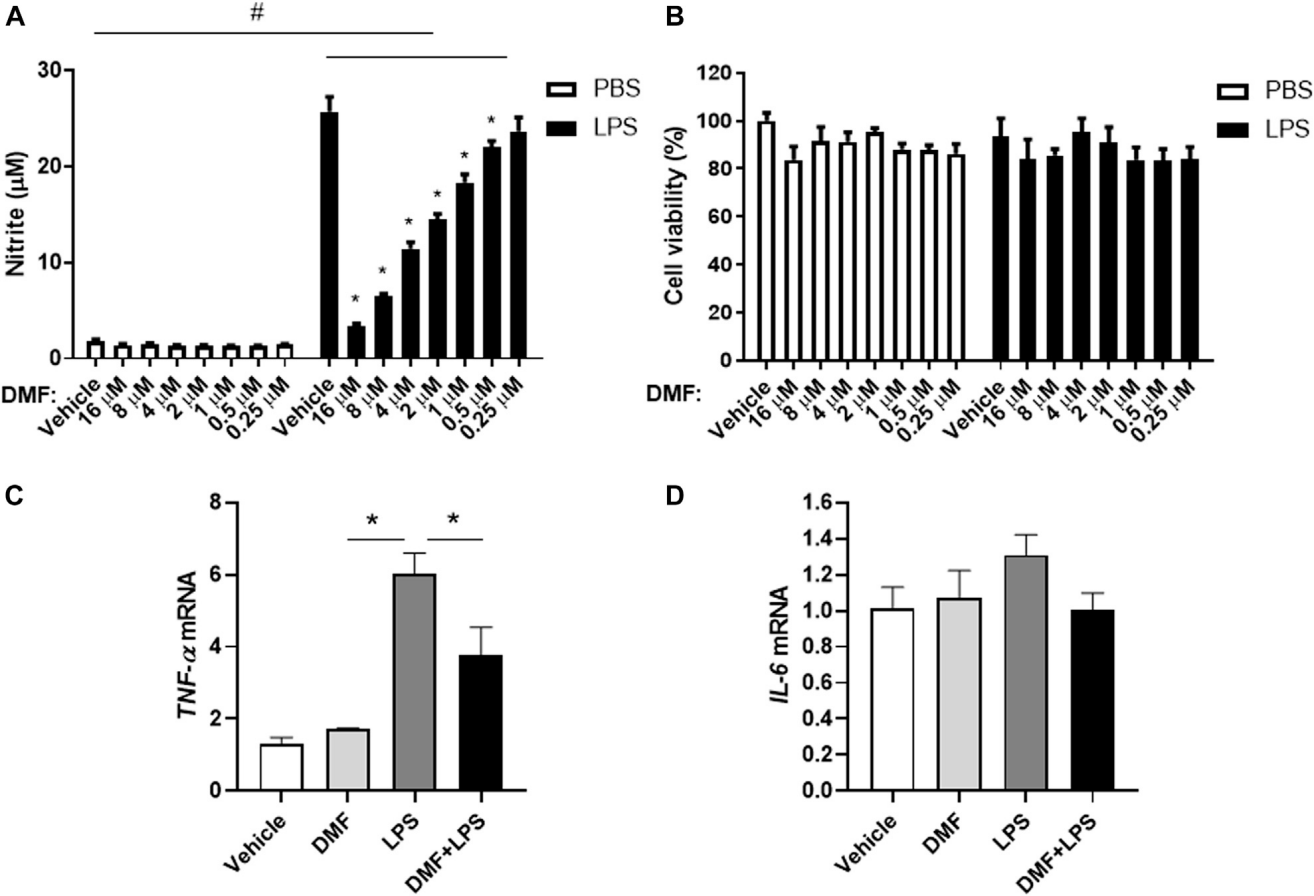
DMF reduces inflammation in microglia. HAPI cells were seeded at 4 × 10^4^ cells/well in a 96-well plate and incubated overnight. Cells were then treated with indicated concentration of DMF with or without LPS. After 24 h, nitric oxide (NO) production was measured by the Griess assay **(A)**, and cell viability was measured by the MTT assay **(B)**.**(C, D)** HAPI cells were seeded at 1 × 10^6^ cells/well in a 6-well plate and incubated overnight. Cells were then treated with 4 μM DMF with or without LPS (100 ng/ml). After 6 h, the mRNA expression levels of tumor necrosis factor-alpha (*TNF-α*) **(C)** and interleukin- 6 (*IL-6*) **(D)** was determined by real-time quantitative polymerase chain reaction (RT-qPCR). Fold changes were calculated as the ratio of expression level in the LPS-only treated group. *n* = 3 independent experiments/group. Data are presented as means ± SEM. *P < 0.05 compared with LPS-only treated group, #*P* < 0.05 compared with vehicle-only treated group from two-way ANOVA with multiple comparison test.

To investigate whether DMF has anti-inflammatory effects in microglia, we measured NO production in HAPI cells after DMF treatment. As shown in Figure 2A, the production of NO was significantly increased by LPS treatment for 24 h; however, the DMF co-treatment led to a dose-dependent reduction in NO production in HAPI cells, without any change in cell viability (Figures 2A,B). In addition, we determined the expression levels of the inflammatory cytokine TNF-α and IL-6 in HAPI cells after DMF treatment (Figures 2C,D). The mRNA levels of *TNF-α* and *IL-6* in HAPI cells, which were significantly increased with 6 h LPS (100 ng/ml) treatment, were significantly reduced in cells treated with 4 μM DMF. We also utilized the immortalized mouse microglial cell line BV-2 to confirm the anti-inflammatory effect of DMF in another microglial cell line. As shown in Supplementary Figure S3, the production of NO and the *IL-1β*, *TNF-α*, and *IL-6* mRNA levels, which were increased by LPS (100 ng/ml) treatment after 24 h, were reduced by 4 μM DMF co-treatment in BV-2 cells.

### Dimethyl Fumarate Induces M2 Polarization and Phagocytosis in Microglia

**FIGURE 3:**
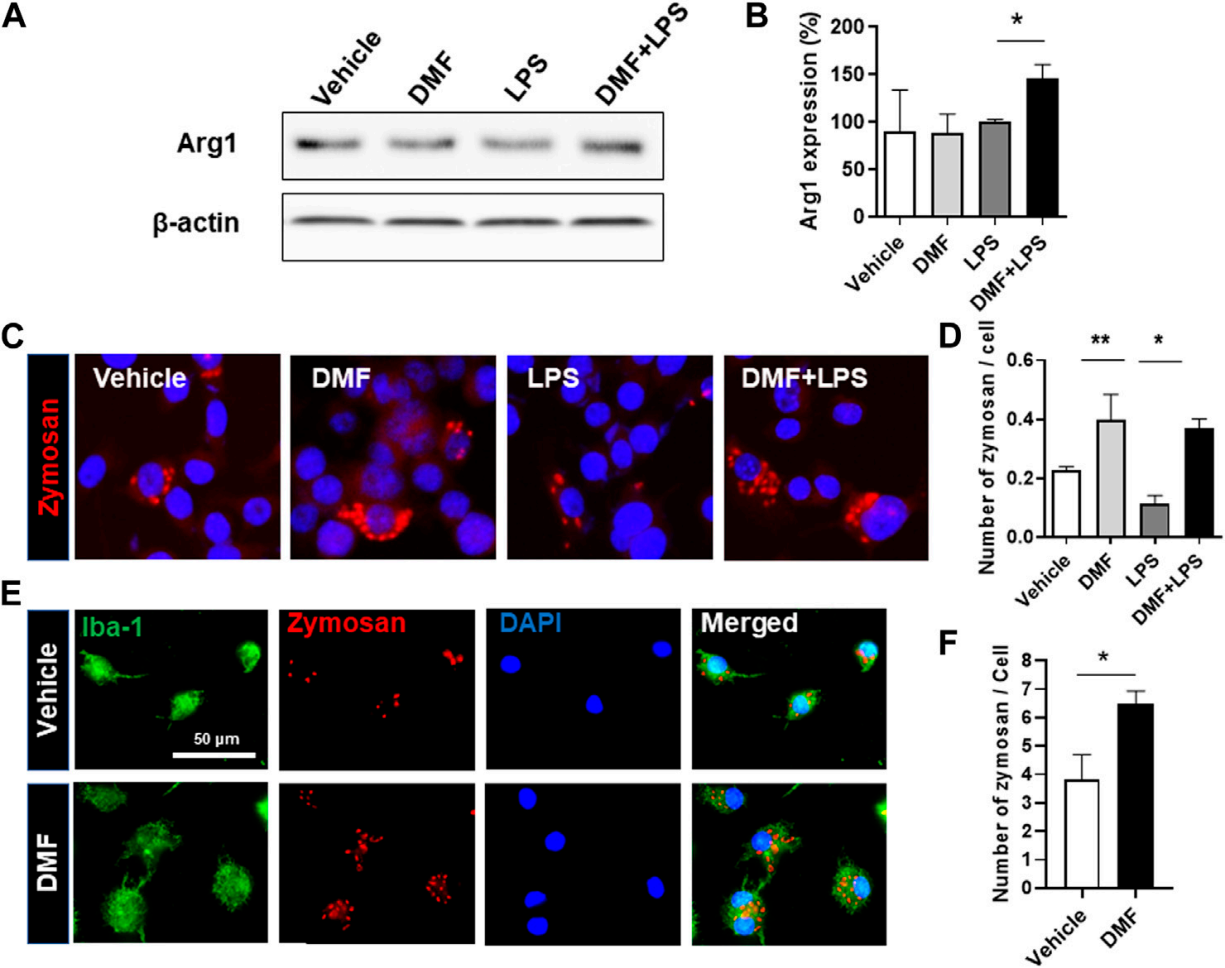
DMF induces the neuroprotective M2 phenotype and phagocytosis in microglia **(A, B)** HAPI cells were seeded at 2.5 × 10^5^ cells/well in a 6-well plate and incubated overnight. Cells were then treated with LPS (100 ng/ml) or DMF (4 μM) plus LPS (100 ng/ml) for 24 h. The expression of arginase-1 protein (Arg1) was determined by Western blot analysis **(B)** Histogram shows the densitometric analysis of Western blots. β-actin was used as the loading control and data were normalized to total β-actin. *n* = 3 independent experiments/group **(C, D)** BV-2 cells were seeded at 5 × 10^4^ cells/well in a 24-well plate and incubated overnight. BV-2 cells were then treated with DMF (4 μM) with or without LPS (100 ng/ml) for 16 h, followed by the addition of opsonized zymosan-red particles into BV-2 cells and then incubated for another 4 h. The phagocytosed zymosan particles were counted and expressed as the number of zymosan particles per cell. A total of 463 control cells, 1117 DMF-treated cells, 990 LPS-treated cells, and 636 DMF + LPS-treated cells were analyzed. Results are representative of two independent experiments. Data are presented as means ± SEM. *P < 0.05, **P < 0.01 compared with LPS-treated mice from ANOVA, Tukey’s multiple comparison test **(E,F)** Primary microglia were seeded at 5 × 10^4^ cells/well in a 24-well plate and incubated overnight. The primary microglia were then treated with DMF (4 μM) for 48 h, followed by the addition of opsonized zymosan-red particles into the primary microglia and then incubated for another 2 h. The primary microglia were stained with the anti-Iba-1 antibody. The phagocytosed zymosan particles were counted and expressed as the number of zymosan particles per Ib1-1 positive microglial cell. A total of 157 control cells and 154 DMF-treated cells were analyzed. *n* = 3 independent experiments/group. Data are presented as means ± SEM. *P < 0.05 from Student’s *t*-test between indicated two groups.

Based on the results showing that DMF treatment induced an anti-inflammatory response in microglia, we next determined whether DMF induced M2 polarization by examining changes in arginase-1 expression, one of the best characterized markers of M2 polarization, in HAPI cells. As shown in Figures 3A,B, the level of arginase-1 protein was significantly increased by 24 h treatment with DMF and LPS compared to 24 h treatment with LPS alone in HAPI cells. While M1 microglia exhibit impaired phagocytic ability, M2 microglia have been demonstrated to maintain efficient phagocytosis (Akhmetzyanova et al., 2019). Thus, we next evaluated the phagocytic ability of primary microglia and BV-2 cells treated with DMF. We found that the phagocytic ability was significantly improved by 16 h treatment with DMF and LPS compared to 16 h treatment with LPS only treated BV-2 cells (Figures 3C,D). In addition, the phagocytosis of fluorescently labeled zymosan particles was significantly higher in the DMF-treated primary microglia compared with the vehicle-treated control primary microglia (Figures 3E,F), suggesting that DMF induced M2 polarization in microglia.

### Dimethyl Fumarate Induces Autophagy in Microglia

**FIGURE 4:**
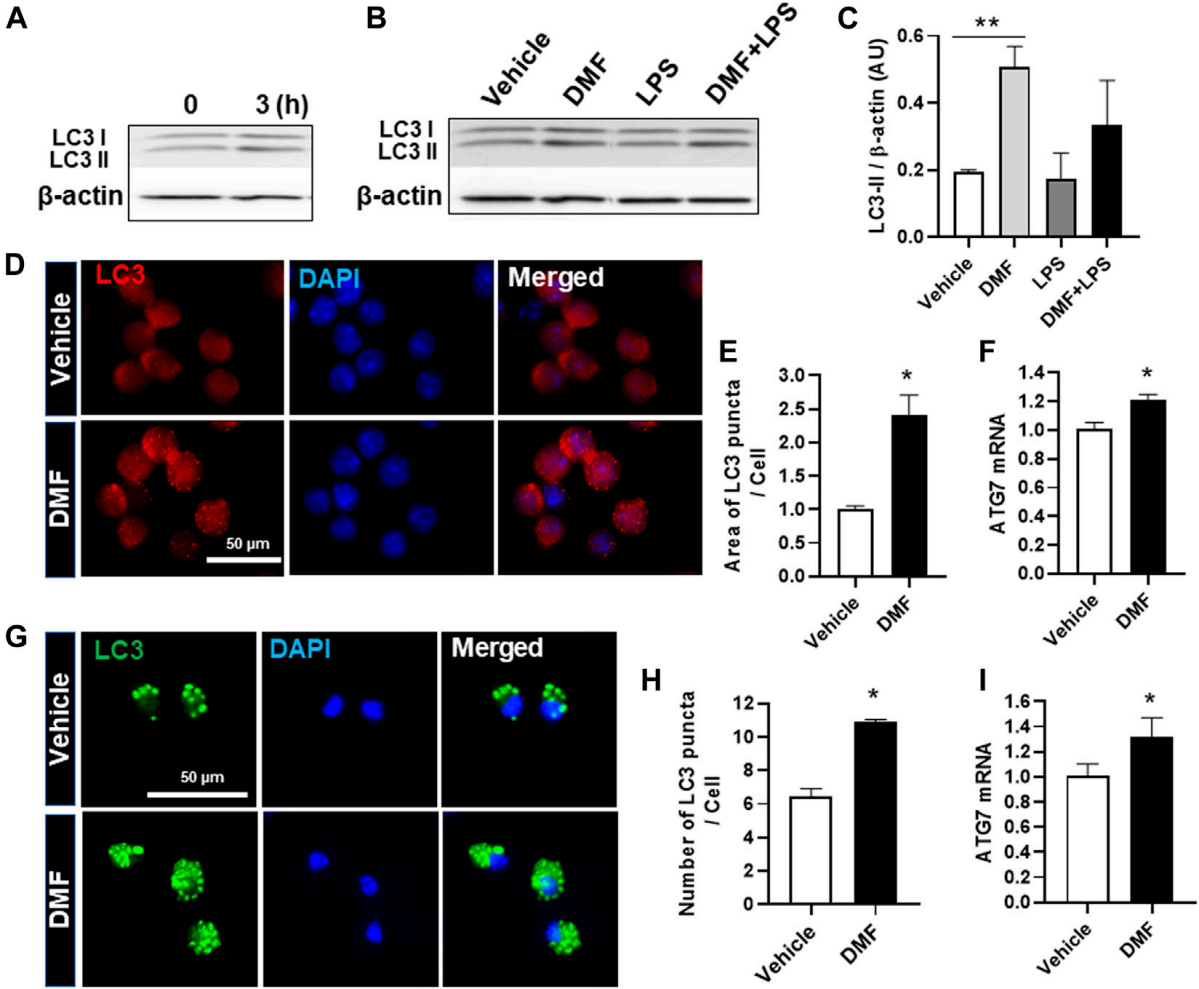
DMF increases autophagy in microglia. HAPI cells were seeded at 2.5 × 10^5^ cells/well in a 6-well plate and incubated overnight **(A)** Cells were then treated with DMF (4 μM) for 3 h **(B)** Cells were treated with LPS (100 ng/ml) or DMF (4 μM) plus LPS (100 ng/ml) for 24 h. The protein levels of LC3-I and LC3-II were determined by Western blot analysis. β-actin was used as the loading control. *n* = 3 independent experiments/group **(C)** Histogram shows the densitometric analysis of Western blots. β-actin was used as the loading control and data were normalized to total β-actin **(D)** HAPI cells were seeded at 2 × 10^4^ cells/well in a 24-well plate and incubated overnight. Cells were then treated with DMF (4 μM) for 24 h and stained with the anti-LC3 antibody **(E)** The punctate area was determined from at least 3 independent images (Vehicle: 29 cells and DMF: 26 cells) using ImageJ and was expressed as the ratio of vehicle controls **(F)** HAPI cells were seeded at 1 × 10^6^ cells/well in a 6-well plate and incubated overnight. Cells were then treated with DMF (4 μM) for 6 h. The mRNA expression levels of *Atg7* was determined by RT-qPCR. Fold changes were calculated as the ratio of expression level in untreated control cells **(G)** Primary microglia isolated from MGC were seeded at 5 × 10^4^ cells/well in a 24-well plate and incubated overnight. Cells were then treated with DMF (10 μM) for 24 h, followed by staining with the anti-LC3 antibody **(H)** The number of LC3 puncta per cell was counted. A total of 27 control cells and 38 DMF-treated cells were analyzed **(I)** MGC were seeded at 2 × 10^5^cells/well in a 12-well plate and incubated overnight. Cells were then treated with DMF (10 μM) for 6 h. The mRNA expression levels of *Atg7* was analyzed by RT-qPCR. Fold changes were calculated as the ratio of expression level in untreated control cells. *n* = 3 independent experiments/group. Data are presented as means ± SEM. *P < 0.05 from Student’s *t*-test for indicated two groups. **P < 0.01 compared with vehicle from ANOVA, Tukey’s multiple comparison test.

Autophagy is a physiological catabolic process which removes unnecessary or dysfunction proteins, and defective autophagy is associated with neuroinflammation. Thus, we next investigated whether DMF induced autophagy in microglia. LC3, which is used as a marker to monitor autophagy, is associated with the formation of autophagosomes and autolysosomes (Wang et al., 2013). To detect autophagy induction in DMF-treated cells, we measured the higher molecular-weight LC3-I and the lower molecular-weight LC3-II by Western blot analysis. As shown in Figure 4A, the expression level of LC3-II was increased at 3 h after DMF treatment in HAPI cells. In addition, the expression of LC3-II was significantly increased by 24-h treatment with DMF compared to vehicle control and showed a tendency of induction in the treatment with DMF and LPS compared with LPS alone in HAPI cells (Figures 4B,C). The measurement of LC3 puncta in HAPI cells by immunofluorescence revealed that the area of LC3 puncta per cell was significantly increased in DMF-treated HAPI cells (Figures 4D,E). The mRNA level of *Atg7*, an essential regulator of autophagosome assembly as one of the autophagy-related genes, was also increased after DMF treatment in HAPI cells (Figure 4F). We also found that the number of LC3 puncta per cell and the *Atg7* mRNA level was significantly increased in MGC treated with DMF (Figures 4G,H,I). These results suggest that DMF induced autophagy in microglia.

### Dimethyl Fumarate Induces Anti-inflammatory Response in Microglia via an Autophagy-dependent Pathway

**FIGURE 5:**
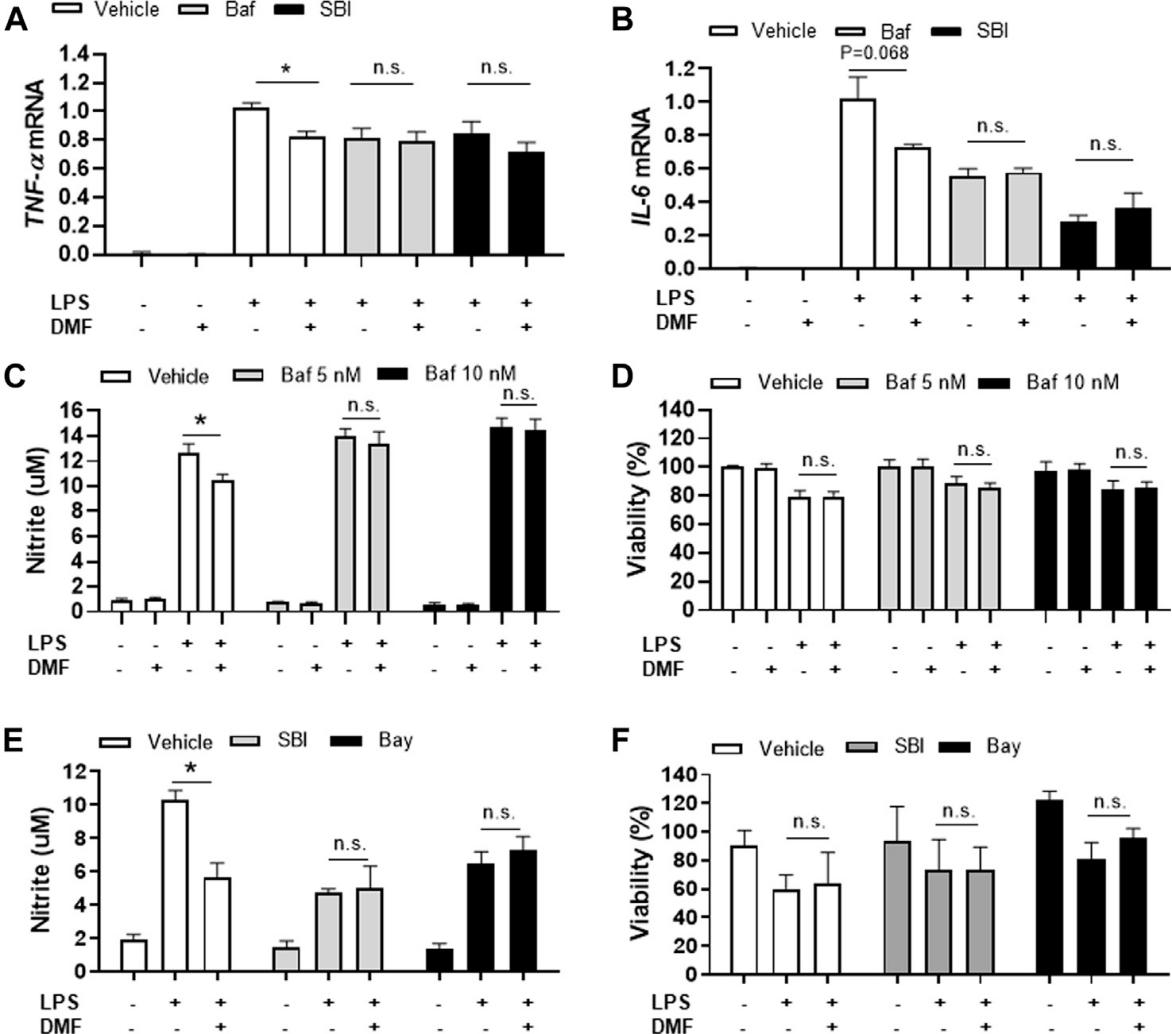
DMF induces anti-inflammatory responses in MGC via an autophagy-dependent pathway **(A, B)** MGC were seeded at 2 × 10^5^ cells/well in a 12-well plate and incubated overnight. Cells were then pretreated with bafilomycin (10 nM, Baf) or SBI-0206965 (5 μM, SBI) for 1 h, followed by treatment with DMF (4 μM) and LPS (1 μg/ml). After 6 h, the mRNA expression levels of *TNF-α* **(A)** and *IL-6* mRNA **(B)** were measured by RT-qPCR. Fold changes were calculated as the ratio of expression level in LPS-treated cells **(C, D)** MGC were seeded at 3 × 10^4^ cells/well in a 96-well plate and incubated overnight. Cells were then pretreated with bafilomycin (5 (gray) or 10 (black)nM) for 1 h, followed by the addition of DMF (4 μM) and LPS (1 μg/ml). After 48 h, NO production was measured by the Griess assay **(C)** and cell viability was measured by the MTT assay **(D) (E, F)** MGC were seeded at 3 × 10^4^ cells/well in a 96-well plate and incubated overnight. Cells were then pretreated with SBI-0206965 (5 μM, gray) or Bay 11–7082 (2.5 μM, Bay, black) for 1 h, followed by treatment with DMF (4 μM) and LPS (1 μg/ml). After 48 h, NO production **(E)** and cell viability **(F)** were measured. *n* = 3 independent experiments/group. Data are presented as means ± SEM. *P < 0.05 compared with LPS treated group, n.s., not significant (*P* > 0.05).

**FIGURE 6:**
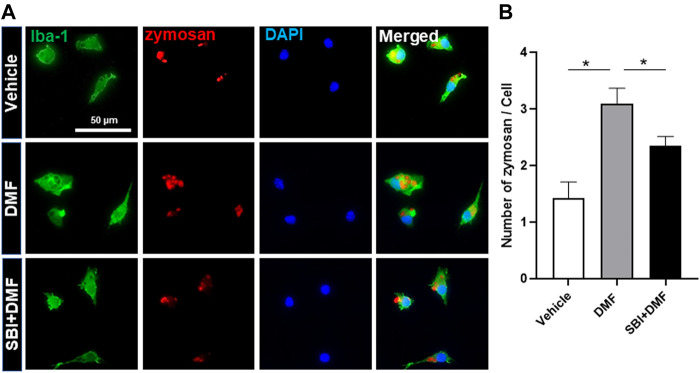
DMF increases phagocytosis via an autophagy-dependent pathway in microglia **(A, B)** Primary microglia were seeded 5 × 10^4^ cells/well in a 24-well plate and incubated overnight. Cells were then pretreated with SBI-0206965 (5 μM) for 1 h, followed by the addition of DMF (10 μM). After 24 h, opsonized zymosan-red particles were added into the cultures which were incubated for 2 h. The primary microglia were stained with the anti-Iba-1 antibody. The phagocytosed zymosan particles were counted and expressed as the number of zymosan particles per cell. The analysis included 78 control cells, 96 cells treated with DMF, and 110 cells treated with DMF plus SBI-0206965. *n* = 3 independent experiments/group. Data are presented as means ± SEM. *P < 0.05 from ANOVA.

Based on our finding that DMF induced autophagy in microglia, we next determined whether DMF-induced autophagy regulated the anti-inflammatory response in microglia by pretreatment of cultures with bafilomycin A1 (Mauvezin and Neufeld, 2015; Redmann et al., 2017) or SBI-0206965 (Martin et al., 2018; Wang et al., 2020), both potent inhibitors of cellular autophagy, for 1 h followed by treatment with DMF for 6 h. As expected, the mRNA levels of the anti-inflammatory cytokines *TNF-α* and *IL-6*, which were significantly increased by LPS treatment, were reduced by DMF treatment. Importantly, bafilomycin A1 and SBI-0206965 completely abrogated the DMF-induced reduction in proinflammatory cytokine production in primary MGC (Figures 5A,B). Additionally, we found that the NO production was significantly inhibited by DMF in LPS-treated MGC; this effect was abolished by pretreatment with the autophagy inhibitors (Figures 5C,E). The cell viability was not different in cells treated with bafilomycin A1 or SBI-0206965 and untreated control cells (Figures 5D,F). In addition, we used the specific NF-κB signaling inhibitor Bay 11–7082, given that DMF has been reported to reduce proinflammatory response through the NF-κB pathway. As shown in Figures 5E,F, Bay 11–7082 completely abrogated the DMF-induced reduction in NO production in primary MGC. These results suggested that not only the NF-κB pathway but also autophagy was involved in DMF-induced anti-inflammatory response in LPS-induced inflammatory response in MGC. In addition, we elucidated whether the DMF-induced phagocytosis was reduced by autophagy inhibition by pretreating cells with the autophagy inhibitor SBI-0206965 for 1 h before treatment with DMF for 24 h. As shown in Figures 6A,B, the significant increase in phagocytosis by DMF in primary microglia was ameliorated by SBI-0206965 pretreatment. These results suggest that DMF-induced autophagy-mediated an anti-inflammatory response in microglia.

## Discussion

Neuroinflammation is a well-known pathophysiological process in many neurodegenerative diseases (Song and Suk, 2017; Stephenson et al., 2018). The crosstalk between neurons and glial cells regulates many physiological and pathological processes (Jha et al., 2018; Zhang et al., 2018; Linnerbauer et al., 2020). Microglia are important players in neuroinflammation as a major immune cell type in the CNS. Studies previously demonstrated that DMF treatment inhibited inflammation by reducing the synthesis of the proinflammatory mediators iNOS, TNF-α, IL-1β, and IL-6 in microglia (Paraiso et al., 2018). In the present study, we also found that DMF suppressed NO production and the mRNA levels of *TNF-α* and *IL-6* in microglial cell lines such as HAPI and BV-2 as well as in primary MGC. M1 microglia exacerbate neuroinflammation, whereas M2 microglia promote tissue repair and provide an anti-inflammatory response (Tang and Le, 2016; Song and Suk, 2017). Intriguingly, M2 microglia exhibit a high level of phagocytosis in the CNS (Cherry et al., 2015). Thus, we determined whether DMF promoted microglial polarization toward the M2 phenotype by investigating the expression of arginase-1 protein as a prototypic M2 marker in mice (Roszer, 2015). Arginase-1-positive microglia have been shown to reduce Aβ plaque number during neuroinflammation in the CNS (Cherry et al., 2015). In the present study, we found that DMF treatment led to a significant increase in arginase-1 protein levels in microglia and induced phagocytosis in primary MGC and BV-2 cells. Phagocytosis is a critical process for maintaining homeostasis in CNS. Thus, understanding how to put the brake on M1 polarization and promote M2 polarization in the CNS may lead to the development of novel therapeutic approaches for targeting neurodegenerative disease. These results suggest that DMF might improve Aβ plaque clearance in the brain and spinal cord and reduce pathology via increased phagocytosis.

Recent findings in diabetic neuropathy demonstrate that glial cells, particularly microglia and astrocytes, are an important source of inflammatory mediators fundamentally involved in the pathogenesis of inflammation and neurodegeneration. Thus, we utilized the mouse model of STZ-induced diabetic neuropathy, in which the spinal cord exhibits microglial reactivation as a sign of neuroinflammation (Kiguchi et al., 2017; Song et al., 2019). In the present study, we found that the spinal dorsal horn showed hypertrophic morphology in mice with STZ-induced diabetic neuropathy, accompanied with an increase in the number of microglia and astrocytes, based on the expression levels of microglial and astrocytic phenotypic markers. These findings are in line with other studies in monkeys with type 2 diabetes and *db/db* mice (Liao et al., 2011; Kiguchi et al., 2017). The suppression of microglial accumulation and activation in the spinal cord are associated with the inhibition of neuropathic pain. In the present study, we found that DMF treatment suppressed gliosis in the spinal cord of mice with STZ-induced diabetic neuropathy, indicating that DMF might be useful for the management of diabetic neuropathy based on its neuroprotective effects. In addition, diabetes-related neurodegeneration is considered to be multifactorial, including hyperglycemia (Jing et al., 2013). Glucose toxicity in the brain has been shown to induce inflammation (Peiro et al., 2016); therefore, we also investigated whether the DMF-induced anti-inflammatory response was due to decreased blood sugar in DMF-treated mice with diabetic neuropathy. The mice with STZ-induced diabetic neuropathy exhibited hyperglycemia compared with the vehicle-administered mice; however, the blood glucose levels did not significantly changed following DMF treatment in the STZ-treated mice (data not shown). These findings indicate that the DMF exerts anti-inflammatory effects in the spinal cord without reducing the blood glucose level in mice with STZ-induced diabetic neuropathy.

Autophagy is essential for the removal of unnecessary cellular constituents and dysfunctional components. Autophagic dysfunction and inflammation are key players in the development of neurodegeneration (Su et al., 2016; Plaza-Zabala et al., 2017). Upregulation of autophagy promotes microglial polarization toward the M2 phenotype by the suppression of M1 markers, including iNOS/NO, TNF-α, and IL-6, and the induction of M2 markers, including arginase-1, Ym1/2, and IL-10. (Jin et al., 2018; Cho et al., 2020). In addition, TNF-α, an important proinflammatory cytokine, inhibits autophagy by impairing autophagic flux through the AKT/mammalian target of the rapamycin signaling pathway in microglia. In the present study, we found that DMF reduced NO production and the expression levels of genes associated with the M1 phenotype, including *TNF-α* and *IL-6*; DMF also induced arginase-1 expression and phagocytosis in microglia, both associated with the M2 phenotype. Thus, we determined whether DMF-induced anti-inflammatory response in microglia was mediated by the upregulation of autophagy and found that DMF induced autophagy in microglia is based on increased expression of LC3, the number and area of LC3 puncta, and *Atg7* mRNA expression level. The formation of autophagosomes is controlled by several ATG proteins such as ATG7 (Strohm and Behrends, 2020). Therefore, we hypothesized that DMF-induced autophagy might play a role in DMF-mediated anti-inflammatory response in microglia. We indeed found that the autophagy inhibitors bafilomycin A1 and SBI-0206965 inhibited the DMF-induced anti-inflammatory response microglia. These results suggest that DMF reduces inflammation via autophagy induction in microglia, which may be beneficial for neuroprotection and treatment of neuroinflammation. However, the role of autophagy in DMF treated microglia is still unclear. Therefore, the mechanism by which DMF induces autophagy in microglia and the level to which it is involved in DMF-induced anti-inflammatory effects in CNS needs to be understood to enable its development as a target of therapeutic approaches for neurodegeneration diseases. In addition, the role of DMF-induced autophagy in neurons or glial cell types, including microglia, astrocytes, and oligodendrocyte, should be investigated in future studies.

In summary, we demonstrated that DMF inhibited reactive microglial activation in the spinal cord and reduced proinflammatory response in microglia via the upregulation of autophagy. These results suggest that the therapeutic potential role of DMF in neurodegenerative diseases may partially involve the induction of autophagy in microglia.

## Data Availability

The original contributions presented in the study are included in the article/Supplementary Material, further inquiries can be directed to the corresponding author.
