# Supplementary material for: Anti-Inflammatory Effects of Dimethyl Fumarate in Microglia via an Autophagy Dependent Pathway
Source: Front Pharmacol. 2021 May 7;12:612981. doi: 10.3389/fphar.2021.612981 (PMC8137969; doi:10.3389/fphar.2021.612981)
Supplement: Supplementary file 1 [file DataSheet1.doc]

**Supplementary Table 1. List of RT-qPCR or RT-PCR primers and sequences.**

| *RT-qPCR* | Sence | Anti-sense |
| --- | --- | --- |
| *Mouse-cyclophilin* | 5’-TGGAGAGCACCAAGACAGACA-3’ | 5’-TGCCGGAGTCGACAATGAT-3’ |
| *Rat-cyclophilin* | 5’- GGTCTTTGGGAAGGTGAAAGAA -3’ | 5’-GCCATTCCTGGACCCAAAA -3’ |
| *Mouse-TNF-α* | 5’-CCAACGGCATGGATCTCAAAGACA-3’ | 5’-AGATAGCAAATCGGCTGACGGTGT-3’ |
| *Rat-*  *TNF-α* | 5’-CAGCCGATTTGCCATTTC A-3’ | 5’-AGGGCTCTTGATGGCAGAGA-3’ |
| *Mouse-*  *IL-6* | 5’-TCCAGTTGCCTTCTTGGGACTGAT-3’ | 5’-AGCCTCCGACTTGTCAAGTGGTAT-3’ |
| *Rat-*  *IL-6* | 5’-TCTCTCCGCAAGAGACTTCCA-3’ | 5’-ATACTGGTCTGTTGTGGGTGG-3’ |
| *Mouse-*  *ATG7* | 5’-CCTGCACAACACCAACAC AC-3’ | 5’-CACCTGACTTTATGGCTTCCC-3’ |
| *RT-PCR* | Sence | Anti-sense |
| *Mouse-GAPDH* | 5’-ACCACAGTCCATGCCATCAC-3’ | 5’-TCCACCACCCTGTTGCTGTA-3’ |
| *Mouse-TNF-α* | 5’-CATCTTCTCAAAATTCGAGTGACAA-3’ | 5’-ACTTGGGCAGATTGACCTCAG-3’ |
| *Mouse-*  *IL-6* | 5’-AGTTGCCTTCTTGGGACTGA -3’ | 5’-TCCACGATTTCCCAGAGAAC-3’ |
| *Mouse-*  *IL-1β* | 5’-GCAACTGTTCCTGAACTC-3’ | 5’-CTCGGAGCCTGTAGTGCA-3’ |

**Supplementary Fig. 1**

**
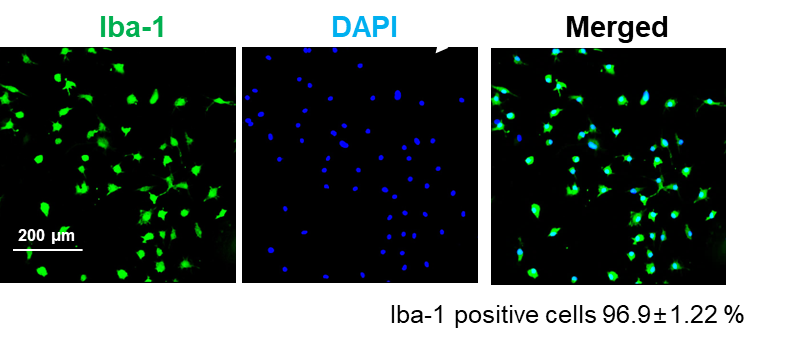
**

**Supplementary Fig. 1.** **The purity of the primary microglia.** Primary microglia isolated from the confluent mouse MGC cultures were plated onto 24 well plates (5 × 10^4^/well) containing poly-D-lysine-coated glass coverslips. Cell were immunostained with anti-Iba-1. The purity of microglia was calculated by Iba-1 positive cells over DAPI (n = 9 mouse fetal brains). Data are presented as the means ± standard deviation (SD).

**Supplementary Fig. 2**

**Example for the quantification of the number of Iba-1 positive microglia count in spinal cord.**


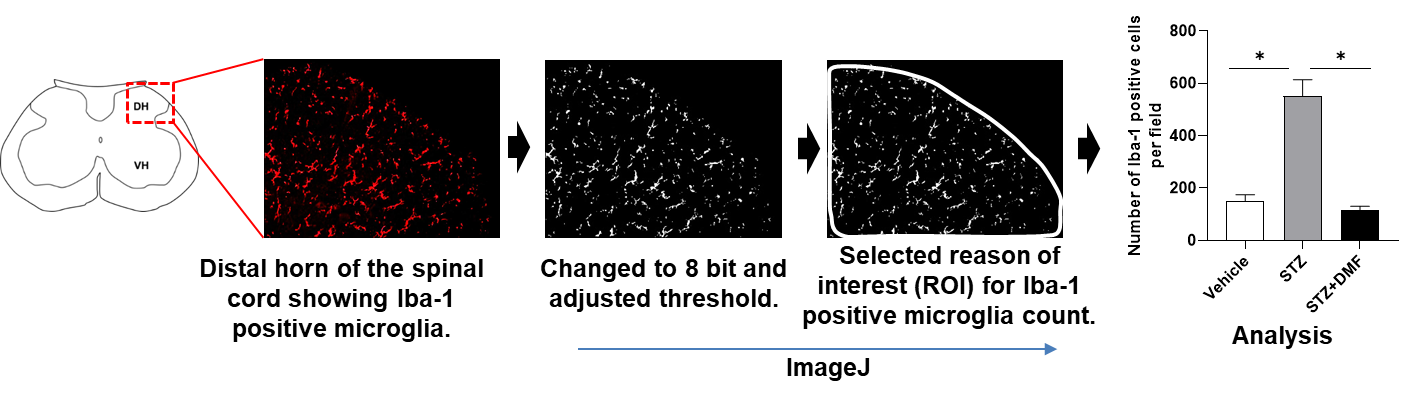


**Explanation for the quantification**

For the quantification of Iba-1, the spinal cord images were outlined the size for the standardized

region of interest (ROI) by ImageJ software, and the percentage of spinal cord area with fluorescence was quantified. The threshold was adjusted and standardized for each images for the accuracy of true protein expression signal for quantification. The percentage was calculated by dividing the pixel

number of protein expression with the pixel number of unfiltered number in the ROI. Three or

four sections per spinal cord tissue was immunostained, averaged and analyzed.

**Supplementary Fig. 3**


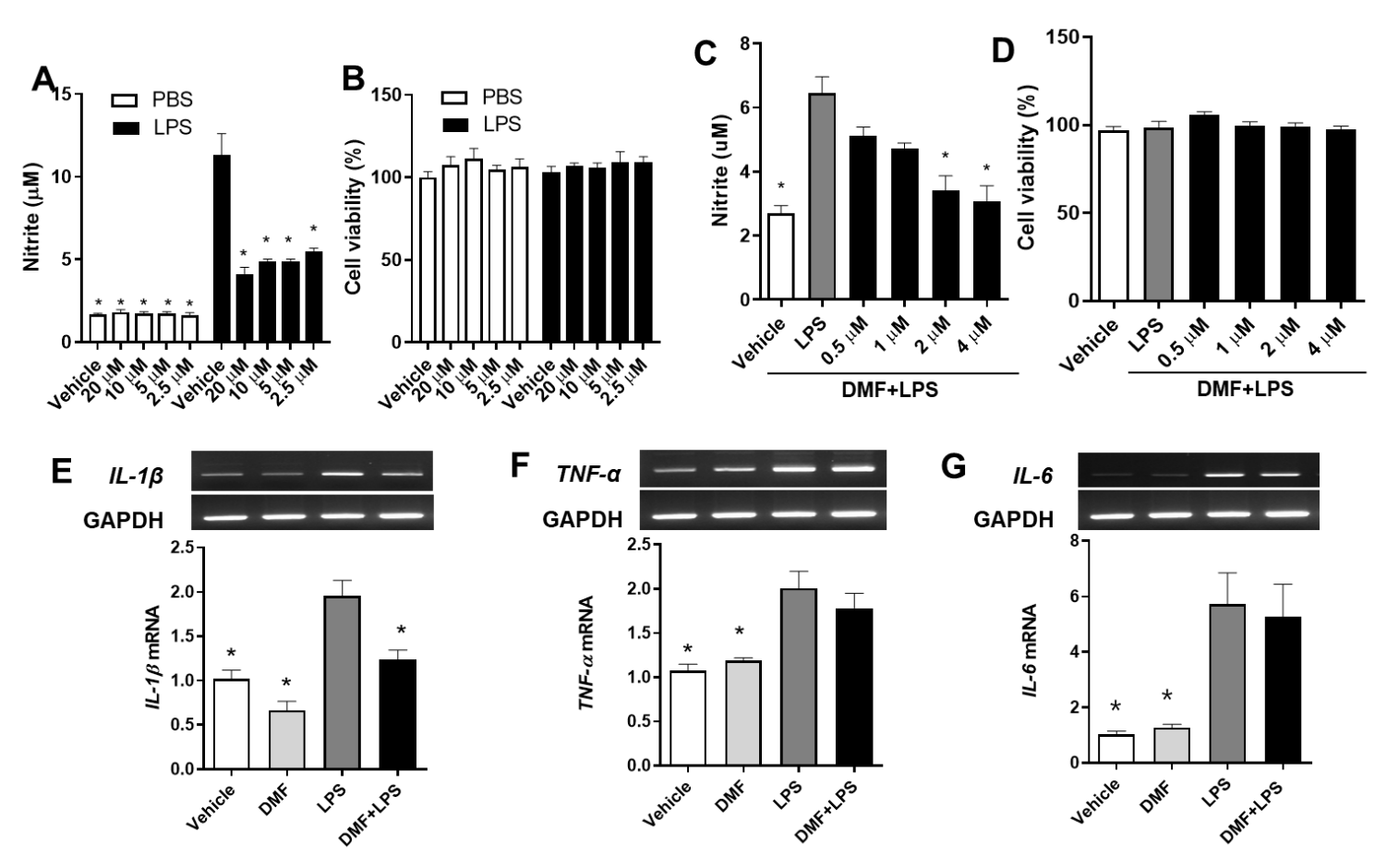


**Supplementary Fig. 3. DMF reduced inflammation in BV-2 microglia.** BV-2 cells were treated with indicated concentrations of DMF or/and LPS (100 ng/ml) for 24 h, NO product was measured by Griess assay (A, C) and cell viability measured by MTT assay (B, D). Cells were treated with DMF (4 μM) or LPS (100 ng/ml). After 24 h, the expression of *IL-1β* (E), *TNF-α* (F), and *IL-6* (G) mRNA was measured by RT-PCR. The fold change was calculated as ratio of the expression level in LPS stimulated cells. Results are representative of 3 independent experiments. Data are means ±SE. * P<0.05 compared with LPS only treated group from ANOVA multicompanies test.
